# Predicting Pharmacokinetic Properties of Potential Anticancer Agents via Their Chromatographic Behavior on Different Reversed Phase Materials

**DOI:** 10.3390/ijms22084257

**Published:** 2021-04-20

**Authors:** Małgorzata Janicka, Anna Mycka, Małgorzata Sztanke, Krzysztof Sztanke

**Affiliations:** 1 Department of Physical Chemistry, Faculty of Chemistry, Institute of Chemical Science, Maria Curie-Skłodowska University, Maria Curie-Skłodowska Sq. 3, 20-031 Lublin, Poland; malgorzata.janicka@poczta.umcs.lublin.pl; 2 Doctoral School of Quantitative and Natural Sciences, Maria Curie-Skłodowska University, Maria Curie-Skłodowska Sq. 3, 20-031 Lublin, Poland; a.e.mycka@gmail.com; 3 Chair and Department of Medical Chemistry, Medical University of Lublin, 4A Chodźki Street, 20-093 Lublin, Poland; 4 Laboratory of Bioorganic Synthesis and Analysis, Chair and Department of Medical Chemistry, Medical University of Lublin, 4A Chodźki Street, 20-093 Lublin, Poland

**Keywords:** micellar chromatography, immobilized artificial membrane chromatography, RP-18e, QSARs, log *K*_p_, log *K*_a,HSA_, log *BB*, Caco-2, *f*_u,brain_, fused azaisocytosine-containing congeners

## Abstract

The Quantitative Structure-Activity Relationship (QSAR) methodology was used to predict biological properties, i.e., the blood–brain distribution (log *BB*), fraction unbounded in the brain (*f*_u,brain_), water-skin permeation (log *K*_p_), binding to human plasma proteins (log *K*_a,HSA_), and intestinal permeability (Caco-2), for three classes of fused azaisocytosine-containing congeners that were considered and tested as promising drug candidates. The compounds were characterized by lipophilic, structural, and electronic descriptors, i.e., chromatographic retention, topological polar surface area, polarizability, and molecular weight. Different reversed-phase liquid chromatography techniques were used to determine the chromatographic lipophilicity of the compounds that were tested, i.e., micellar liquid chromatography (MLC) with the ODS-2 column and polyoxyethylene lauryl ether (Brij 35) as the effluent component, an immobilized artificial membrane (IAM) chromatography with phosphatidylcholine column (IAM.PC.DD2) and chromatography with end-capped octadecylsilyl (ODS) column using aqueous solutions of acetonitrile as the mobile phases. Using multiple linear regression, we derived the statistically significant quantitative structure-activity relationships. All these QSAR equations were validated and were found to be very good. The investigations highlight the significance and possibilities of liquid chromatographic techniques with three different reversed-phase materials and QSARs methods in predicting the pharmacokinetic properties of our important organic compounds and reducing unethical animal testing.

## 1. Introduction

The use of various chromatographic techniques in supporting the drug discovery process and in physicochemical research has become quite extensive. The search for new biologically active substances, considered as potential drugs or plant protection products, is an important task in modern science. The goals are to improve people’s quality of life and their life expectancy and to increase agricultural productivity while ensuring diversity and protecting the environment. One way to achieve the above goal is to synthesize new compounds that have the desired properties. Since the 19th century, it has been known that the properties of chemical substances are closely related to their molecular structures. The intensive development of the Quantitative Structure-Activity Relationships (QSARs) method began in the 1960s and continues today [1,2,3]. In this method, searches are conducted to identify the multidimensional relationships that exist between the biological properties and structural parameters for a group of congeneric compounds. The derived mathematical model can be extended to new compounds with similar structures and used to predict their biological properties. In this way, it is possible to design new molecules that have the desired properties. The model becomes the basis for making decisions concerning the synthesis of new compounds, which allows researchers to limit the time and cost associated with their research. In addition, the interpretation of a mathematical model can lead to an overall model of a given biological property, which provides information that can be used to obtain the optimal design of desired chemical substances.

The relationship between solute activity and the parameters describing its molecular properties can be reported as a multiple linear regression (MLR) [2,3,4]:

*Activity* = *aA* + *bB* + *cC* + … + const = *f*(lipophilic, electronic, steric properties) (1)

where *a*, *b*, *c*, and so on are the correlation factors. The molecular descriptors (*A*, *B*, *C*, …) relating to the lipophilic, electronic, and steric properties of the molecule can be determined experimentally or evaluated in silico. Currently, there are many software products on the market that allow such calculations, e.g., HyperChem, ACD/ChemSketch, ACD/LADME, and SciLogP.

The lipophilicity of a bioactive compound is usually expressed by the logarithm of the partition coefficient in an n-octanol/water system and is either measured experimentally by the “shake-flask” method (log *P*_o/w_) or evaluated in silico using different algorithms (fragment, atomic, molecular, or combined atomic-fragment) from molecular structures. Retention parameters, especially log *k*_w_ values, measured by a column or by planar Reversed-Phase Liquid Chromatography (RPLC), are the most popular, and they are accepted as chromatographic lipophilicity descriptors by the Organization of Economic Co-operation and Development. In addition, liquid chromatography techniques are very popular as indirect in vitro methods for the determination of lipophilicity [5,6,7]. The chromatographic methods used to assess lipophilicity have significant advantages, e.g., simplicity and reduced experimental time, good reproducibility, process automation, no need for quantitative analysis, and small amounts of samples required. The chromatographic methods also provide independent measurements of the low solubility of the compound and measurements of impurities or degradation products. However, some limitations of the RPLC method have also been noted. The most important of these limitations are: (1) Insufficient modeling of the n-octanol-water system for structurally diverse compounds, (2) the effect of the pore size and possible interactions with the residual silanol groups on the surface that do not occur in the n-octanol-water partitioning system, (3) time-consuming isocratic measurements, and (4) a limited working range of pH. Some solutions have been developed in the last few decades to overcome these limitations. First, it is worth mentioning the novel types of columns that were designed to mimic the n-octanol-water system (e.g., polymeric reversed-phase columns (PLRPs) or polymer-based columns) or to mimic biological partitioning, e.g., immobilized artificial membranes (IAMs) or columns with immobilized cholesterol, human or rat serum albumins, glycoproteins, and others [8,9,10]. Modifying the mobile phase is another solution. Such possibilities offer Micellar Liquid Chromatography (MLC) using surfactants as components of the mobile phase [5,11,12,13] and ionic liquids as effluents [14]. A specific type of micellar chromatography is Biopartitioning Micellar Chromatography (BMC), in which Brij 35 and a low concentration of alcohol, if necessary, are added to the mobile phase. The hydrophilic/hydrophobic nature of the surfactant in the modified stationary phase structurally resembles the ordered array of the hydrocarbon chains in the biomembranes. In addition, the surfactant and alcohol that are present in the mobile phase provide the opportunity for hydrogen bonds to form. This technique is usually referred to as Biopartitioning Micellar Chromatography [15,16] due to its similarity with biological barriers and extracellular fluids. An important advantage of MLC is that it meets the recommendations of green chemistry by limiting the consumption of organic solvents [17].

The most important biological properties of substances considered as potential drugs are their ability to bind blood proteins (albumin and alpha-1-acid glycoprotein); penetrate biological barriers, e.g., the blood–brain barrier (BBB); permeate the skin; and perform intestinal absorption. When entering into plasma, most compounds bind rapidly to the constituents of blood, but the concentration of a free drug is responsible for the pharmacological activity, safety, and distribution of the tissue. So, the extent of protein binding in plasma affects the pharmacokinetic characteristics of a compound, i.e., its clearance, volume of distribution, half-life, drug-drug interaction, and pharmacological efficacy. Agents intended to interact with the central nervous system must be able to cross the BBB, and satisfactory transport through the blood–brain barrier is an essential prerequisite for a potential drug to affect the central nervous system. However, in order to avoid side effects, the agents that act peripherally should not cross the BBB. In both cases, the permeability of the BBB must be known and should be evaluated at the earliest possible stage of testing. Intestinal absorption is particularly important for oral medications that are transported into the blood via the intestinal tract [11,18]. Conventionally, biomimetic descriptors require animal testing (e.g., rats, dogs, monkeys, or humans). In vivo tests are extremely unethical and inhumane. They also require significant financial outlays and time that are inconsistent with the results that are achieved. Over the past few decades, along with the rapid development of new computational discoveries, combinatorial chemistry, and high-throughput biological research, it has become possible to accelerate the selection of “ideal” drug candidates for further development. If the structure of a compound is known, then it is possible to predict its lipophilic, biological, and physicochemical properties. However, in silico methods do not provide reliable results for substances with more complex structures. Compared with conventional methods, chromatography using biomimetic systems, recognized as an in vitro technique, is becoming increasingly popular.

The compounds that were tested 1–19 belong to three anticancer active classes of structurally related small molecules (Table 1) that share the same privileged heterocyclic scaffold [19,20,21]. Moreover, two classes of compounds possess isosteric groups such as the isopropyl in 1–6 and trifluoromethyl in 7–14. Two novel sets of fused azaisocytosine-containing congeners 1–6 and 7–14 are antimetabolites that possess the elucidated mechanism of their antiproliferative action (by caspase activation). They were synthesized in our laboratory and patented. These azaisocytosine-containing congeners were described in our earlier paper in which their medical anticancer utility was also mentioned [19]. Most of the title molecules exhibited more potent cytotoxicity in human cancer cells than a clinically approved anticancer agent, pemetrexed, and also revealed similar or more protective effects than that of ascorbic acid and Trolox in an ex vivo model of rat erythrocytes exposed to oxidants. The compounds 8, 10, 11, 17, and 19 exhibited the clearly higher antiproliferative effects in cancer cells than in normal cells [19,20,21]. In addition, the compounds 7, 8, and 10–12 were shown to be safer for the early life stages of *Danio rerio* than pemetrexed [22]. Due to the important pharmacological activity, all these organic substances require more thorough and extensive research on modeling their pharmacokinetic properties.

In our present research, we used the following protocol: (**1**) The in vitro determination of chromatography-based lipophilicity parameters of the tested compounds using reversed-phase materials capable of imitating pharmacokinetic and partitioning processes in biological systems and an end-capped ODS column; (**2**) the in silico calculation of structural and electronic descriptors (topological polar surface area, polarizability, and molecular weight); (**3**) the in silico calculation of partition coefficients (log *P*) and pharmacokinetic properties (e.g., log *BB*, *f*_u,brain_, log *K*_p_, log *K*_a,HSA_, and Caco-2) affecting drug-like properties of the tested compounds from molecular structures using an ACD/Percepta software; (**4**) the establishment and validation of new QSAR models, which make it possible to predict the pharmacokinetic properties (such as log *BB*, *f*_u,brain_, log *K*_p_, log *K*_a,HSA_, and Caco-2) of the title compounds on the basis of their experimental lipophilicity parameters and structural and electronic descriptors; and (**5**) the visualization of correlations between the dependent solute properties obtained from newly constructed QSAR models and those established in silico.

## 2. Results

### 2.1. Chromatographic Data

There are several theories that describe the effect of the concentration of the surfactant in the effluent on the retention of the solute in MLC [23]. The following Foley’s equation is best known in lipophilicity studies [24]:

(2) $$\frac{1}{k}=\frac{1}{k_{m}}+\frac{K_{AM}}{k_{m}}\left[M\right]$$

where [*M*] is the total concentration of the surfactant in the mobile phase minus *CMC*; *K_AM_* is the constant that describes solute-micelle binding; and *k_m_* is the solute retention parameter at the micellar concentration of zero, i.e., when the concentration of the surfactant monomer is equal to *CMC*. The *K_AM_* and *k_m_* parameters can be evaluated from the slope and intercept of the experimental 1/*k* vs. [*M*] relationships.

Equation (2) describes a linear dependence between decreasing retention and increasing micelle concentration. This equation is valid for aqueous solutions of surfactant or mobile phases with the same concentrations of the organic modifier. The micellar retention parameter, log *k*_m_, is considered analogous to the log *k*_w_ parameter evaluated in RPLC. According to the information presented above, this parameter is considered to be a lipophilicity descriptor, and Equation (2) is a simple way to indirectly determine the lipophilic properties of compounds. It is postulated that retention in micellar chromatography depends on hydrophobic (lipophilic), electronic, and steric features of the compounds in a similar way as has been noted concerning pharmacokinetic phenomena. The additional similarity results from the fact that the phospholipids, cholesterol, fatty acids, and triglycerides included in the extracellular and intracellular fluids also form micelles with proteins.

In our investigations, the micellar retention factors were used to calculate the log *k*_m_ parameters using Equation (2) (Table 2). For all of the compounds 1-19, the relationships of 1/*k* vs. [*M*] were obtained with very good linearity, confirming that Foley’s equation correctly describes the effect of the concentration of the surfactant in the effluent on the retention of the solute. In our studies, the micellar log *k*_m_ parameters for three pharmacologically active sets of compounds 1-6, 7-14, and 15-19 and the log *k* factors for solutes 15-19 obtained on IAM and end-capped ODS stationary phases were determined experimentally. All these retention factors, together with the log *k*_w_ values for compounds 1-14 obtained in our earlier investigations on IAM and end-capped ODS stationary phases [19], were used as lipophilicity descriptors in the QSARs methodology to predict the pharmacokinetic descriptors of the compounds that were tested.

### 2.2. Establishment of Quantitative Structure-Activity Relationships

In the QSARs methodology, we used the experimentally derived lipophilicity (chromatographic parameters: log *k*_m_, log *k*_w,IAM_, and log *k*_w,ODS_), structural (topological polar surface area *TPSA*, molecular weight *MW*), and electronic (polarizability *α*) descriptors as independent variables. These values were used to predict different pharmacokinetic parameters (dependent variables) evaluated for tested compounds (Table 2). Table 3 shows the quantitative structure-activity relationships (expressed as Equations (3)–(22)) that were established. The relationships were validated, and the results are presented in Table 4.

**Figure 1:**
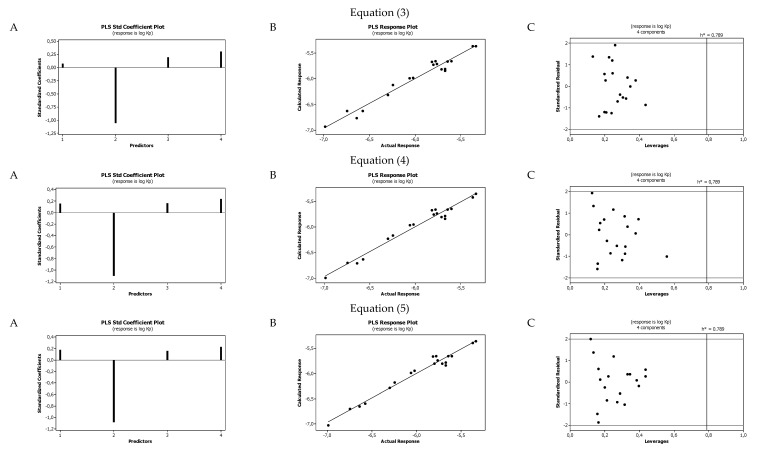
Standardized coefficients (**A**), the correlation between actual (ACD/Percepta) and predicted (Equations (3)–(5)) log *K*_p_ parameters (**B**), and the Williams plots of Equations (3)–(5) (**C**).

The statistical parameters allowed us to evaluate the derived QSAR equations as being very good. There were no significant cross-correlations between the parameters that characterized the substances, i.e., the values of the variance inflation factor (*VIF*) were significantly lower than 5. The diagrams presented in Figure 1A show the standard coefficients of Equations (3)–(5) established for the log *K*_p_ as an example. The remaining diagrams are presented in Figures S1A–S4A in the Supplementary Material. They explain both the direction and strength of the impact of a given structural descriptor on the calculated biological parameter. The correlations shown in Figure 1B illustrate the relationships between the log *K*_p_ values calculated with the ACD/Percepta software (actual response) and those predicted by the QSARs models (calculated response) that were developed (Equations (3)–(5)). The remaining diagrams are presented in Figures S1B–S4B in the Supplementary Material. The applicability domain (AD) of the developed regression models was also evaluated and visualized as the Williams plots (Figure 1C and Figures S1C–S4C in the Supplementary Material). AD is a theoretical region in physicochemical space (the response and chemical structure space) for which a QSAR model should make predictions with a given reliability. The warning leverage limits (h* = 0.789 and 0.632) were calculated using the following equation:

(23) $$h^{*}=\frac{3(k+1)}{n}$$

where *k* is a number of descriptors used in the MLR model and *n* is the number of compounds in the dataset. The Williams plot can be used for graphical detection of outliers (*h* > h*) [25]. The results proved that the obtained models are valid within the domain in which they were developed.

### 2.3. Assessing the Risk of Undesired Effects

Many of potential molecular pharmaceutics cannot be subjected to clinical trials on humans due to the risk of serious adverse effects. Hence, in silico tools such as the OSIRIS Property Explorer (http://www.organic-chemistry.org/prog/peo/ accessed on 25 March 2021), recommended by Food and Drug Administration, are helpful in qualitative prediction of serious adverse side effects in the early stage of the drug development process. For all the investigated compounds 1-19 considered as potential anticancer agents, no risk of mutagenicity, tumorigenicity, and irritating effects were predicted. In addition, no risk (for compounds 1-14) or medium risk (for compounds 15-19) of reproductive effects was found. This is as expected due to the lack of “genotoxicophore” fragments in the tested molecules. The results are shown in Table S1 in the Supplementary Material.

## 3. Discussion

The lipophilic properties of compounds increase their binding to human serum albumin and to the lipids contained in biological membranes. In the investigations, the lipophilic properties of our compounds were described based on their chromatographic retention (log *k*_m_, log *k*_w,ODS_, and log *k*_w,IAM_), and the positive effect of these parameters on the log *K*_p_, log *K*_a,HSA_, log *BB*, Caco-2, and *f*_u,brain_ values was obtained (Figure 1, Figures S1–S4 in the Supplementary Material). Taking into account the standardized coefficients, the lipophilicity had a similar, moderate impact on the above parameters. The same effect was found for *α*, i.e., the polarizability of the molecules. This parameter increased the strength of the van der Waals interactions between the solutes and the albumin or lipids molecules [26]. Thus, the polarizability of the molecule increased the values of log *K*_p_, log *K*_a,HSA_, and log *BB*. Polarizability increased the values of log *K*_p_ and log *K*_a,HSA_ similarly to or slightly more than lipophilicity. In the case of log *BB*, polarizability seemed to be the dominant positive factor. We observed no effect of polarizability on the values of Caco-2 and *f*_u,brain_ (Equations (12)–(17)). The positive effect of molecular weight (*MW*) on the values of log *K*_p_, log *K*_aHSA_, and Caco-2 could be explained by the partition mechanism of the permeation of the tested substances through biological membranes as well as human serum albumin. Similarly, Abraham et al. [27] obtained the positive effect of molecular size on the permeability through the skin. This relationship is a reflection of the correlation between the size of the molecules and lipophilicity. In addition, molecular size has a negative correlation with diffusivity in biomembranes, confirming that the effects of partitioning are more dominant than the effects of diffusion [28].

The polar molecular surface area (*PSA*) is defined as the surface area occupied by the nitrogen and oxygen atoms and the polar hydrogens bonded to these heteroatoms. The penetration of substances through biological barriers decreases when the hydrophilic part of its surface increases. *PSA* has been used extensively as a molecular descriptor in the studies of drug transport properties, such as intestinal absorption [29], BBB penetration [30], and membrane permeability [28,31,32]. Topological surface area (*TPSA*), a convenient measure of the polar surface area, was introduced by Ertl et al. [33] as the effect of the additive fragment method and is extremely popular in medicinal chemistry [34] for predicting the properties of ADME. In our research, we observed a significant negative impact of *TPSA* on log *K*_p_, log *BB*, and the Caco-2 parameters (Equations (3)–(5) and (9)–(14)). The increase of the polar surface area decreased the permeability through the skin, permeation of the blood–brain barrier, and intestinal permeability.

The factors that increase the substances that bind to serum albumin and lipids cause a simultaneous reduction of the unbound fraction in the brain, *f*_u,brain_. The equations derived in our studies (Equations (15)–(17)), Figure S4 in the Supplementary Material) show that *f*_u,brain_ decreased with increasing lipophilicity and molecular weight (*MW*) but increased with the hydrophilicity (*TPSA*) of the compound. Polarizability had a negligible effect on the *f*_u,brain_ values.

In RPLC, the standard lipophilicity descriptors are the log *k*_w_ parameters evaluated for water (buffer) as the mobile phase. In the case of micellar chromatography, the log *k*_m_ values were used (Equation (2)), corresponding to the mobile phase without any “free” surfactant molecules. In general, the determination of these parameters is time-consuming and requires multiple measurements using different mobile phases. Nevertheless, the quantities determined in this way are more reliable and similar to the partitioning parameter, log *P*. Frequently, in practice, the chromatographic parameters measured with mobile phases that contain an organic modifier can also be used to evaluate lipophilicity. Most often, experimental data are used that were measured with columns imitating biological systems, such as artificial membranes, immobilized cholesterol, and others. In our studies, we obtained very good linear correlations between the log *k* values obtained in MLC for mobile phases with different concentrations of Brij 35, i.e., 0.15 mol/L, 0.10 mol/L, 0.125 mol/L, and 0.075 mol/L (Table 2). The correlation factors of these relationships were in the range of 0.902–0.942. Therefore, we decided to use the log *k* parameters measured in one micellar effluent to derive the quantitative structure-activity relationships. We chose the values measured in the mobile phase composed of 0.1 mol/L of surfactant Brij 35, i.e., log k_0.1_. For this mobile phase, the retention of individual substances was not too high (log *k* values in the range of 0.279–1.67). At the same time, the flow of effluent through the column was not associated with high pressure. Appropriate equations (Equations (18)–(22)) and statistics are presented in Table 3 and Table 4. In the statistical evaluation, these equations were similar and almost as good as those derived for the log *k*_m_, log *k*_w,IAM_, and log *k*_w,ODS_ parameters. The results indicate the effectivity of micellar chromatography and its predictive ability in assessing the properties of bioactive substances. This technique also provided the advantage of being able to mimic biopartitioning systems. On the basis of the chromatographic measurements performed in one system with a micellar mobile phase, our results show that there is a high probability that the pharmacokinetic properties of the tested compounds can be predicted accurately.

## 4. Materials and Methods

### 4.1. Reagents and Materials

Isopropanol, acetonitrile (HPLC grade), and polyoxyethylene lauryl ether (Brij 35) (for synthesis) were supplied from Merck (Lublin, Poland). Citric acid and Na_2_HPO_4_ (both pure) were purchased from POCh (Lublin, Poland). Deionized water was produced using the Direct-Q3 UV system (Millipore, Warsaw, Poland).

### 4.2. Instrumental

Shimadzu Vp (Shimadzu, Izabelin, Polska) liquid chromatographic system was used in HPLC measurements. It was equipped with an LC 10AT pump, SPD 10A UV–Vis detector, SCL 10A system controller, CTO-10 AS chromatographic oven, and Rheodyne injector valve with a 20 μL loop. As the stationary phases, 3 different revered-phase materials were applied: Spherisorb ODS-2 column, 125 × 4 mm i.d., 5 μm (Merck, Lublin, Poland), Regis IAM.PC.DD2 column, 100 × 4.6 mm i.d., 10 μm (Morton Grove, Illinois, USA), and Purosphere RP-18e column, 125 × 4 mm i.d., 5 μm (Merck, Lublin, Poland).

### 4.3. Chromatographic Conditions

In the MLC technique with an ODS-2 column, buffered Brij 35 mixtures (0.15; 0.125, 0.10, and 0.075 mol/L) with 7% (*v*/*v*) addition of isopropanol were used as mobile phases. The buffer was prepared from 0.01 mol/L solutions of Na_2_HPO_4_ and citric acid, and the pH 7.4 value was fixed before mixing with an organic modifier. The flow rate was 1 mL/min. Buffered acetonitrile mixtures were used as effluents with the IAM column. Acetonitrile concentration, expressed as a volume fraction, was changed in the range of 0.2–0.5, with the constant step of 0.1. The flow rate was 1.3 mL/min. Acetonitrile concentration was changed in the range of 0.3–0.6 with the RP-18e column, with the constant step of 0.1 and flow rate of 0.1 mL/min. As solutes tested there were used 19 newly designed structurally related compounds. Samples were dissolved in acetonitrile c.a. 0.005 mg/mL. The compounds were detected under UV light at λ_max_ 254 nm. All measurements were carried out at a constant temperature (25 °C). The dead time values were measured from non-retained compound (e.g., sodium chloride) peaks. All reported *k* values are the average of at least 3 independent measurements.

### 4.4. In Silico Calculations

Molecular weight (*MW*), topological polar surface area (*TPSA*) and polarizability (α) of the tested compounds (as independent variables), as well as pharmacokinetic parameters characterizing their distribution between the blood and brain (log *BB*), fraction unbounded in brain (*f*_u,brain_), water–skin permeation (*K*_p_), binding to human plasma proteins (log *K*_a,HSA_), intestinal permeability (Caco-2) (as dependent variables), and the logarithms of n-octanol/water partition coefficient (logs *P*), were evaluated by ACD/Percepta software (Łodź, Poland). In this software, the logs *P* and pharmacokinetic descriptors are calculated from Abraham solvation parameters (i.e., the McGowan volume, polarizability/dipolarity, hydrogen bond basicity (accepting ability) and hydrogen bond acidity (donating ability), excess molar refraction, etc.), according to the concept of LSERs (linear solvation energy relationships) [35].

The risk of adverse effects of the investigated compounds was evaluated by the OSIRIS software, which is available online: http://www.organic-chemistry.org/prog/peo/ (accessed on 25 March 2021). This in silico tool uses the final datasets from the Registry of Toxic Effects of Chemical Substances (RTECS) database containing 7504 mutagenic, 2841 tumorigenic, 2372 irritant, and 3570 reproductive effective substances, as well as 3343 pharmaceutics as a control set. The qualitative prediction result encoded in green, yellow, and red indicates no risk, medium risk, and high risk of undesired effects, respectively.

### 4.5. Statistical Analysis

Linear regression (LR), multiple linear regression (MLR), and leave-one-out cross validation (LOOcv) were done employing the statistical software Minitab 16 (Minitab Inc., State College, PA, USA).

## 5. Conclusions

Two-dimensional QSAR methodology was successful in modeling pharmacokinetic properties, i.e., the distribution between the blood and brain (log *BB*), the unbounded fraction in the brain (*f*_u,brain_), water–skin permeation (log *K*_p_), binding to human plasma proteins (log *K*_a,HSA_), and intestinal permeability (Caco-2) of fused azaisocytosine-containing congeners. Various liquid chromatography techniques were used to characterize all the title compounds regarded as promising drug candidates. Micellar parameters (log *k*_m_) and log *k*_w_ values measured on an artificial membrane (IAM) and on an end-capped ODS column were compared as lipophilicity descriptors and applied in the QSARs methodology. Apart from the chromatography-derived lipophilicity, the quantitative structure-activity relationships included both structural and electronic descriptors related to drug-like properties, i.e., topological polar surface area, molecular weight, and polarizability of the investigated molecules. All the derived QSAR equations were evaluated statistically and validated as being very good. It should be noted that the QSAR models that were developed revealed a high predictive ability and therefore provided reliable predictions in modeling the pharmacokinetic properties of the title molecules. All models used for prediction of the dependent solute property linked the retention parameters on MLC, IAM, and ODS with additional molecular descriptors related to drug-like properties. All the dependent pharmacokinetic properties obtained on the basis of QSAR equations were compared with those calculated in silico and were statistically validated as being very good. Applicability domains of the developed regression models were evaluated and visualized. The investigations highlight the significance and possibilities of combined chromatographic techniques and QSARs methods in modeling important pharmacokinetic properties of our structurally related small molecules and reducing unethical animal testing. The micellar liquid chromatography technique made it possible to achieve a significant reduction in the time and cost of the experiments and also reduced the consumption of organic reagents. The results presented in this study will be particularly useful in further, more extensive in vivo research of the title compounds that are being considered as potential drugs.

## Figures and Tables

**Table 1:** The compounds tested and their structure, molecular weight (*MW*), topological polar surface area (*TPSA*), polarizability (*α*), pharmacokinetic parameters (log *K*_p_, log *K*_a,HSA_, log *BB*, Caco-2, *f*_u.brain_), and lipophilicity (log *P*).

No	R	Structure	*MW* [g/mol]	*TPSA* [A^2^]	*α* [A^3^]	log *K*_p_	log *K*_a,HSA_	log *BB*	Caco-2 E06 [cm/s]	*f*_u,brain_	log *P*
1	H	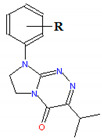	256.30	48.27	29.30	−6.061	4.80	0.230	187	0.52	2.089
2	4-CH_3_		270.33	48.27	31.06	−5.711	4.86	0.423	217	0.32	2.701
3	2-Cl		290.75	48.27	31.13	−5.800	5.09	0.339	215	0.33	2.608
4	3-Cl		290.75	48.27	31.13	−5.645	5.07	0.384	221	0.28	2.757
5	4-Cl		290.75	48.27	31.13	−5.817	5.16	0.328	208	0.38	2.559
6	3,4-Cl_2_		325.19	48.27	32.95	−5.370	5.44	0.473	230	0.21	3.250
7	H	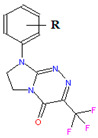	282.22	48.27	25.85	−6.021	4.96	0.102	196	0.44	1.661
8	2-CH_3_		296.25	48.27	27.61	−5.672	5.01	0.290	220	0.27	2.273
9	4-CH_3_		296.25	48.27	27.61	−5.672	5.01	0.290	220	0.27	2.273
10	2-OCH_3_		312.25	57.50	28.16	−6.245	5.03	0.063	195	0.43	1.647
11	2-Cl		316.67	48.27	27.68	−5.762	5.29	0.211	220	0.27	2.151
12	3-Cl		316.67	48.27	27.68	−5.603	5.31	0.264	221	0.26	2.345
13	4-Cl		316.67	48.27	27.68	−5.778	5.33	0.194	218	0.29	2.131
14	3,4-Cl_2_		351.11	48.27	29.50	−5.332	5.64	0.345	235	0.14	2.828
15	H	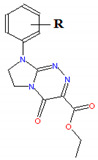	286.29	74.57	30.27	−6.989	5.04	−0.243	134	0.73	0.931
16	4-CH_3_		300.31	74,57	32.02	−6.640	5.07	−0.051	169	0.59	1.548
17	3-Cl		320.73	74,57	32.09	−6.575	5.25	−0.090	179	0.55	1.605
18	4-Cl		320.73	74.57	32.09	−6.746	5.35	−0.151	170	0.59	1.401
19	3,4-Cl_2_		355.18	74,57	33.91	−6.300	5.60	0.000	202	0.42	2.132

**Table 2:** Chromatographic data obtained for all the tested compounds from MLC technique (*k*, log *k*_m_) with the ODS-2 column and Brij 35 as the effluent component and on IAM (log *k*_w,IAM_) and ODS (log *k*_w,ODS_) columns; *k*_m_—parameters calculated from Equation (2); R^2^—coefficient of determination calculated for Equation (2).

No	*k* 0.075 M Brij 35	*k* 0.1 M Brij 35	*k* 0.125 M Brij 35	*k* 0.15 M Brij 35	log *k*_m_	*R* ^2^	log *k*_w,IAM_	log *k*_w,ODS_
1	13.73	12.03	10.47	10.53	1.29	0.8851	0.76 [19]	1.59 [19]
2	25.51	21.45	18.25	17.34	1.68	0.9662	1.05 [19]	2.01 [19]
3	8.98	8.18	7.46	7.20	1.07	0.9725	0.65 [19]	1.42 [19]
4	39.84	31.65	24.39	22.52	2.28	0.9732	1.49 [19]	2.38 [19]
5	41.53	33.76	26.18	24.10	2.22	0.9754	1.39 [19]	2.32 [19]
6	60.81	46.76	34.01	29.82	2.82	0.9854	1.67 [19]	2.68 [19]
7	14.11	12.22	10.72	10.02	1.37	0.9862	0.94 [19]	1.92 [19]
8	6.50	5.97	5.52	5.28	0.92	0.9889	0.76 [19]	1.81 [19]
9	25.67	21.44	17.64	16.14	1.81	0.9870	1.25 [19]	2.16 [19]
10	5.89	5.40	5.04	4.79	0.88	0.9929	0.67 [19]	1.65 [19]
11	10.61	9.36	8.27	7.66	1.24	0.9947	0.88 [19]	1.86 [19]
12	36.90	29.09	22.17	19.83	2.52	0.9855	1.66 [19]	2.43 [19]
13	41.71	32.28	25.32	22.45	2.49	0.9911	1.58 [19]	2.40 [19]
14	59.61	44.28	32.68	28.56	2.70	0.9887	2.29 [19]	2.96 [19]
15	2.09	1.90	1.81	1.84	0.38	0.8467	0.48	1.21
16	4.49	4.04	3.81	3.75	0.74	0.9084	1.93	2.80
17	10.12	8.72	8.00	8.00	1.12	0.8604	1.73	2.62
18	9.94	8.48	7.53	7.75	1.12	0.8467	1.12	1.92
19	24.39	18.98	14.97	14.81	1.83	0.9117	3.36	3.24

**Table 3:** The established Quantitative Structure-Activity Relationships (*n*—number of observations, *R*^2^—coefficient of determination, *sd*—standard deviation, *F*-value, *p* —probability value, *VIF*—variance inflation factor).

No of Equation	QSAR Equations	*n*	*R* ^2^	*sd*	*F*	*p*	*VIF*
(3)	log *K*_p_ = −7.137(0.935) + 0.272(0.079)log *k*_m_ − 0.025(0.006)*TPSA* + 0.041(0.030)*α* + 0.003(0.003)*MW*	19	0.9593	0.108	83	0.000000	<4.4
(4)	log *K*_p_ = −6.109(0.540) + 0.110(0.053)log *k*_w,IAM_ − 0.044(0.002)*TPSA* + 0.035(0.013)*α* + 0.005(0.001)*MW*	19	0.9677	0.096	106	0.000000	<2.7
(5)	log *K*_p_ = −6.250(0.428) + 0.157(0.058)log *k*_w,ODS_ − 0.043(0.002)*TPSA* + 0.034(0.012)*α* + 0.004(0.001)*MW*	19	0.9723	0.089	123	0.000000	<2.3
(6)	log *K*_a,HSA_ = 2.383(0.244) + 0.063(0.022)log *k*_m_ + 0.010(0.007)*α* + 0.008(0.001)*MW*	19	0.9368	0.064	75	0.000000	<1.3
(7)	log *K*_a,HSA_ = 2.412(0.4220) + 0.018(0.043)log *k*_w,IAM_ + 0.008(0.010)*α* + 0.008(0.001)*MW*	19	0.9031	0.079	47	0.000000	<2.6
(8)	log *K*_a,HSA_ = 2.356(0.3500) + 0.019(0.049)log *k*_w,ODS_ + 0.008(0.009)*α* + 0.008(0.001)*MW*	19	0.9028	0.079	46	0.000000	<2.1
(9)	log *BB* = −0.041(0.155) + 0.017(0.020)log *k*_m_ − 0.019(0.002)*TPSA* + 0.043(0.007)*α*	19	0.9554	0.048	108	0.000000	<2.6
(10)	log *BB* = 0.051(0.150) + 0.033(0.017)log *k*_w,IAM_ − 0.020(0.001)*TPSA* + 0.041(0.006)*α*	19	0.9630	0.043	130	0.000000	<1.8
(11)	log *BB* = −0.005(0.133) + 0.049(0.020)log *k*_w, ODS_ − 0.020(0.001)*TPSA* + 0.040(0.06)*α*	19	0.9673	0.041	148	0.000000	<1.8
(12)	Caco-2 E06 = 159.92(28.86) + 3.76(4.77)log *k*_m_ − 1.90(0.29)*TPSA* + 0.47(0.13)*MW*	19	0.8799	9.723	37	0.000000	<2.5
(13)	Caco-2 E06 = 203.38(31.52) + 10.08(4.16)log *k*_w,IAM_ − 2.11(0.18)*TPSA* + 0.34(0.12)*MW*	19	0.9101	8.413	51	0.000000	<2.2
(14)	Caco-2 E06 = 183.81(26.42) + 12.63(4.90)log *k*_w,ODS_ − 2.04(0.17)*TPSA* + 0.35(0.11)*MW*	19	0.9133	8.260	53	0.000000	<2
(15)	*f*_u,brain_ = 0.739(0.147) − 0.014(0.024)log *k*_m_ + 0.012(0.001)*TPSA* − 0.003(0.001)*MW*	19	0.9139	0.050	53	0.000000	<2.5
(16)	*f*_u,brain_ = 0.585(0.174) − 0.036(0.023)log *k*_w,IAM_ + 0.013(0.001)*TPSA* − 0.003(0.001)*MW*	19	0.9243	0.046	61	0.000000	<2.2
(17)	*f*_u,brain_ = 0.643(0.144) − 0.050(0.027)log *k*_w,ODS_ + 0.012(0.001)*TPSA* − 0.003(0.001)*MW*	19	0.9286	0.045	65	0.000000	<2
(18)	log *Kp* = −6.780(0.470) + 0.079(0.122)log *k*_0.1_ − 0.043(0.005)*TPSA* + 0.042(0.017)*α* + 0.006(0.001)*MW*	19	0.9590	0.109	82	0.000000	<4.6
(19)	log *K*_a,HSA_ = 2.292(0.255) + 0.106(0.043)log *k*_0.1_ + 0.010(0.007)*α* + 0.008(0.001)*MW*	19	0.9300	0.067	67	0.000000	<1.2
(20)	log *BB* = −0.052(0.155) + 0.033(0.043)log *k*_0.1_ − 0.019(0.002)*TPSA* + 0.042(0.007)*α*	19	0.9551	0.048	107	0.000000	<3
(21)	Caco-2 E06 = 153.28(26.64) + 13.46(8.48)log *k*_0.1_ − 1.75(0.28)*TPSA* + 0.43(0.11)*MW*	19	0.8929	9.182	42	0.000000	<2.3
(22)	*f*_u,brain_ = 0.762(0.142) − 0.038(0.045)log *k*_0.1_ + 0.012(0.001)*TPSA* − 0.001(0.001)*MW*	19	0.9158	0.049	55	0.000000	<2.3

**Table 4:** Statistical parameters of Equation (3)–(22): PRESS—predicted residual sum of squares, MSE—mean square error, cv—cross validated.

Equation	Adjusted *R*^2^	*PRESS*	*MSE*	*R*^2^cv	*PRESS*cv	*MSE*cv
Equation (3)	0.9476	0.279	0.012	0.9593	0.279	0.012
Equation (4)	0.9585	0.220	0.009	0.9677	0.220	0.009
Equation (5)	0.9644	0.170	0.008	0.9368	0.093	0.004
Equation (6)	0.9241	0.093	0.004	0.9368	0.093	0.004
Equation (7)	0.8837	0.153	0.006	0.9031	0.153	0.006
Equation (8)	0.8834	0.147	0.006	0.9028	0.147	0.006
Equation (9)	0.9465	0.052	0.002	0.9554	0.052	0.002
Equation (10)	0.9556	0.043	0.002	0.9630	0.043	0.002
Equation (11)	0.9608	0.036	0.002	0.9673	0.036	0.002
Equation (12)	0.8559	2468	94.54	0.8799	2468	94.54
Equation (13)	0.8921	1827	70.8	0.9101	1827	66.54
Equation (14)	0.8960	1824	68.23	0.9133	1824	68.23
Equation (15)	0.8967	0.058	0.002	0.9139	0.058	0.002
Equation (16)	0.9091	0.049	0.002	0.9243	0.049	0.002
Equation (17)	0.9143	0.044	0.002	0.9286	0.044	0.002
Equation (18)	0.9473	0.284	0.012	0.9590	0.248	0.012
Equation (19)	0.9160	0.111	0.005	0.9300	0.111	0.005
Equation (20)	0.9462	0.054	0.002	0.9551	0.054	0.002
Equation (21)	0.8715	2311	84.32	0.8929	2311	84.32
Equation (22)	0.8989	0.058	0.002	0.9158	0.058	0.002

## Data Availability

Not applicable.

## Supplementary Materials

The following are available online at https://www.mdpi.com/article/10.3390/ijms22084257/s1. Figure S1. Standardized coefficients (A), the correlation between actual (ACD/Percepta) and predicted (Equations (6)–(8)) log *K*_a,HSA_ parameters (B), and the Williams plots of Equations (6)–(8) (C). Figure S2. Standardized coefficients (A), the correlation between actual (ACD/Percepta) and predicted (Equations (9)–(11)) log *BB* parameters (B), and the Williams plots of Equations (9)–(11) (C). Figure S3. Standardized coefficients (A), the correlation between actual (ACD/Percepta) and predicted (Equations (12)–(14)) Caco-2 parameters (B), and the Williams plots of Equations (12)–(14) (C). Figure S4. Standardized coefficients (A), the correlation between actual (ACD/Percepta) and predicted (Equations (15)–(17)) *f*_u,brain_ parameters (B), and the Williams plots of Equations (15)–(17) (C). Table S1. Risk assessment of adverse side effects by OSIRIS Property Explorer for the investigated compounds 1-19.

## Sample Availability

Samples of all the investigated fused azaisocytosine-containing congeners 1-19 are available from the authors.
