# Supplementary material for: Predicting Pharmacokinetic Properties of Potential Anticancer Agents via Their Chromatographic Behavior on Different Reversed Phase Materials
Source: Int J Mol Sci. 2021 Apr 20;22(8):4257. doi: 10.3390/ijms22084257 (PMC8072580; doi:10.3390/ijms22084257)
Supplement: Supplementary file 1 [file ijms-22-04257-s001.zip › ijms-1179480-supplementary.pdf]

International Journal of
Molecular Sciences

MDPI

Supplementary material

Predicting pharmacokinetic properties of potential anticancer agents *via* their chromatographic behaviour on different reversed phase materials

Małgorzata Janicka [1], Anna Mycka [2], Małgorzata Sztanke [3*] and Krzysztof Sztanke [4*]

[1] Department of Physical Chemistry, Faculty of Chemistry, Institute of Chemical Science, Maria Curie-Skłodowska University, Lublin, Poland
[2] Doctoral School of Quantitative and Natural Sciences, Maria Curie-Skłodowska University, Lublin, Poland
[3] Chair and Department of Medical Chemistry, Medical University of Lublin, Lublin, Poland
[4] Laboratory of Bioorganic Synthesis and Analysis, Chair and Department of Medical Chemistry, Medical University of Lublin, Lublin, Poland
* Correspondence: malgorzata.sztanke@umlub.pl (MS), krzysztof.sztanke@umlub.pl (KS); Tel.: +48 814486195

International Journal of
Molecular Sciences

MDPI

Equation (6)

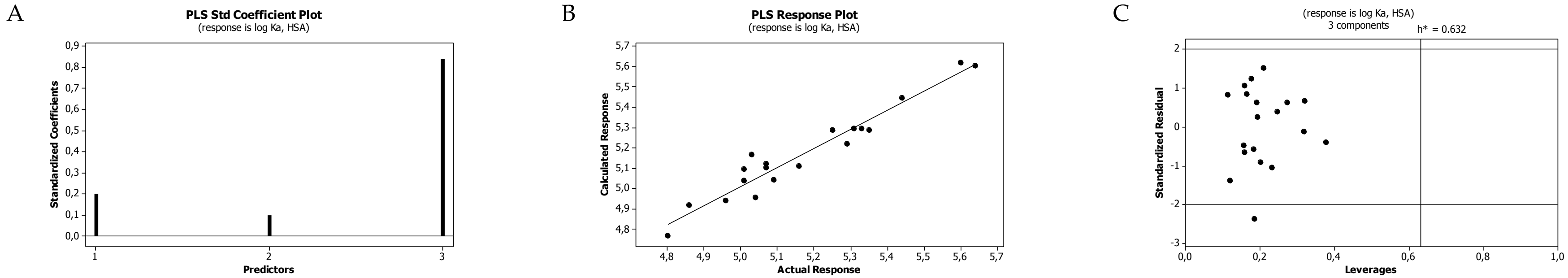

Equation (7)

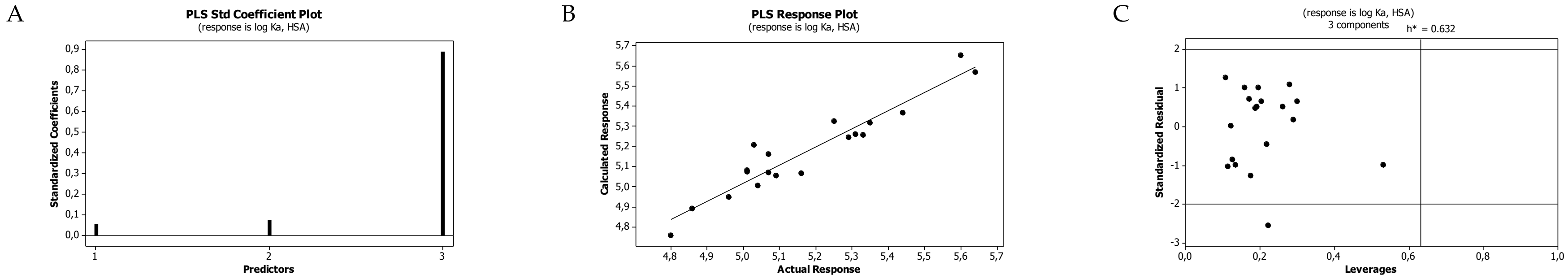

Equation (8)

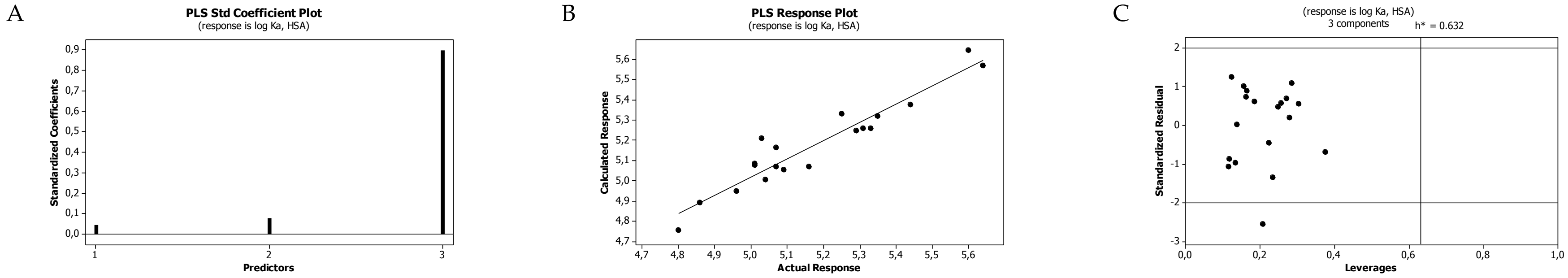

Figure S1. Standardized coefficients (A), the correlation between actual (ACD/Percepta) and predicted (Eqs 6-8) log $K_{a,\ HSA}$ parameters (B), and the Williams plots of Eqs 6-8 (C).

Equation (9)

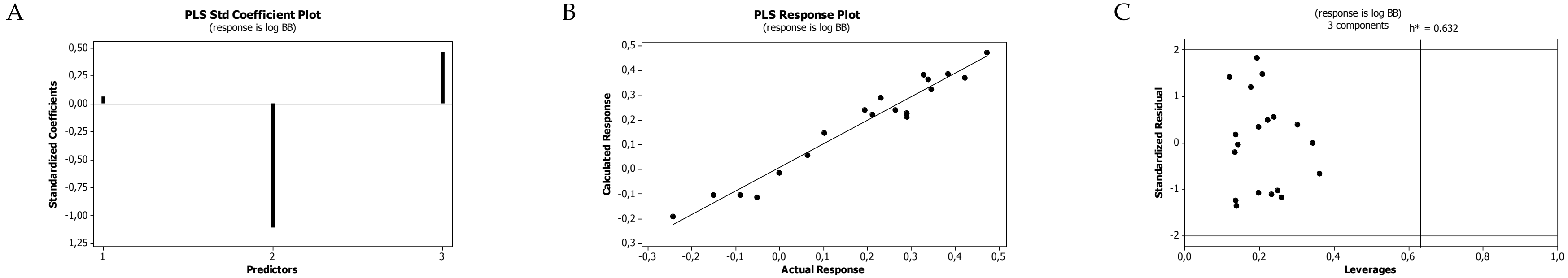

Equation (10)

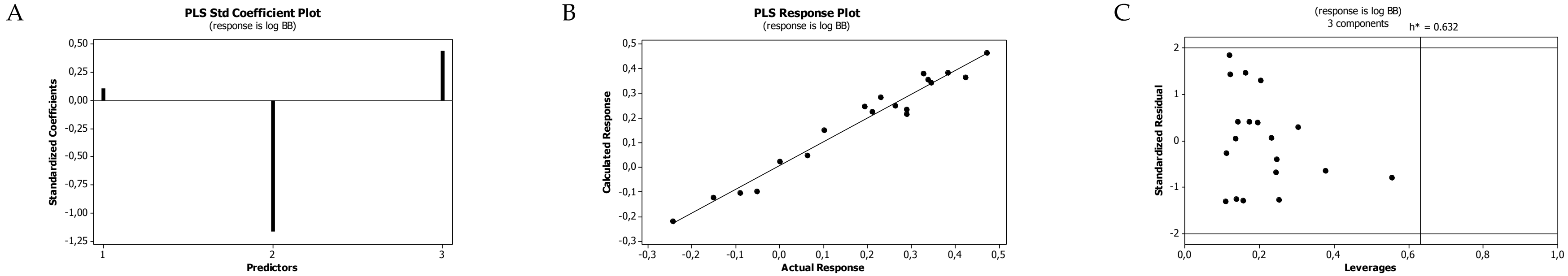

Equation (11)

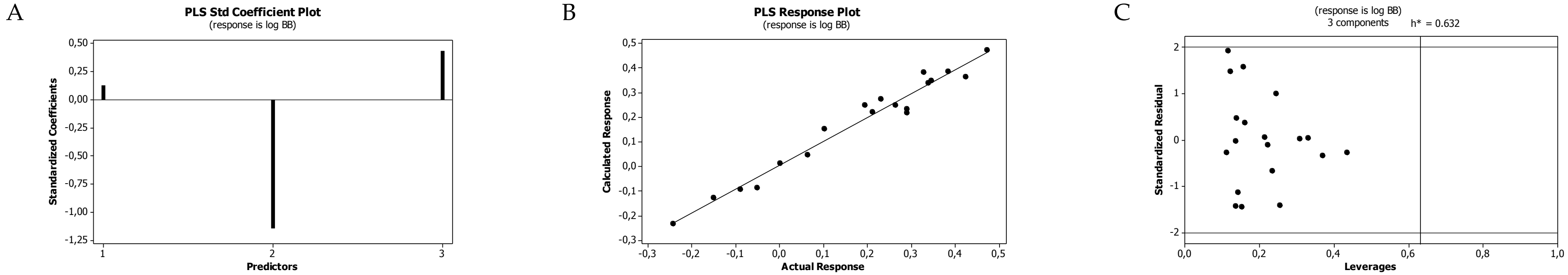

Figure S2. Standardized coefficients (A), the correlation between actual (ACD/Percepta) and predicted (Eqs 9-11) log *BB* parameters (B), and the Williams plots of Eqs 9-11 (C).

Equation (12)

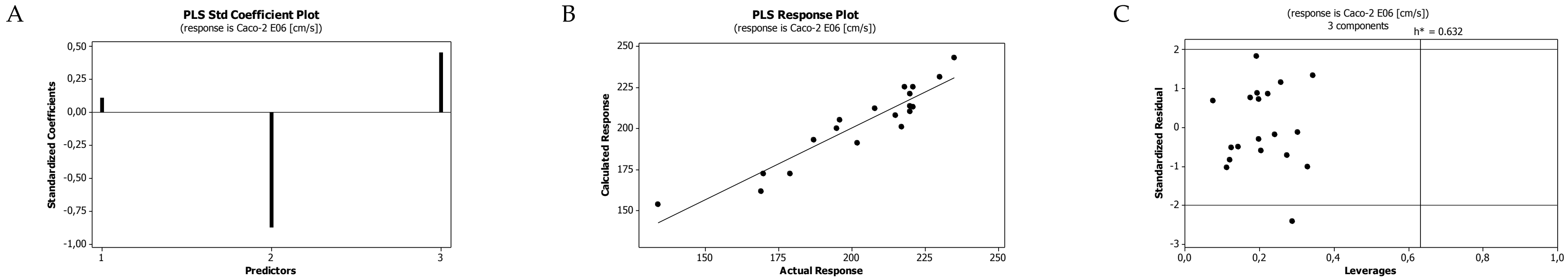

Equation (13)

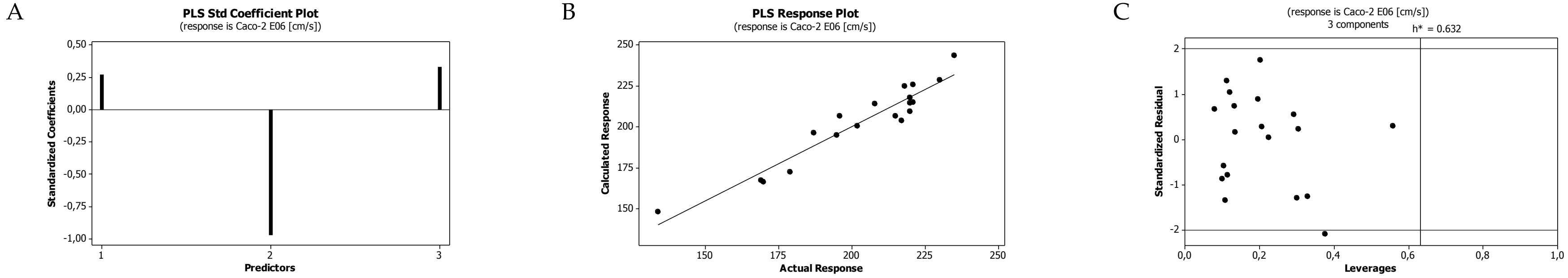

Equation (14)

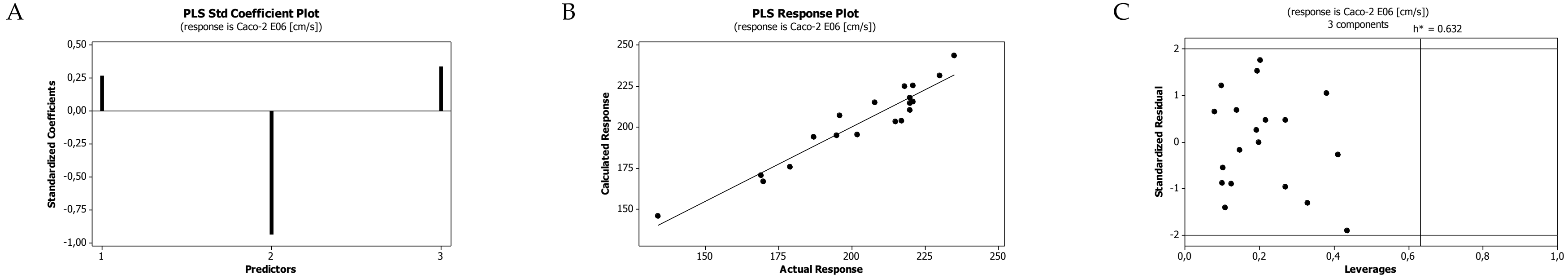

Figure S3. Standardized coefficients (A), the correlation between actual (ACD/Percepta) and predicted (Eqs 12-14) Caco-2 parameters (B), and the Williams plots of Eqs 12-14 (C).

Equation (15)

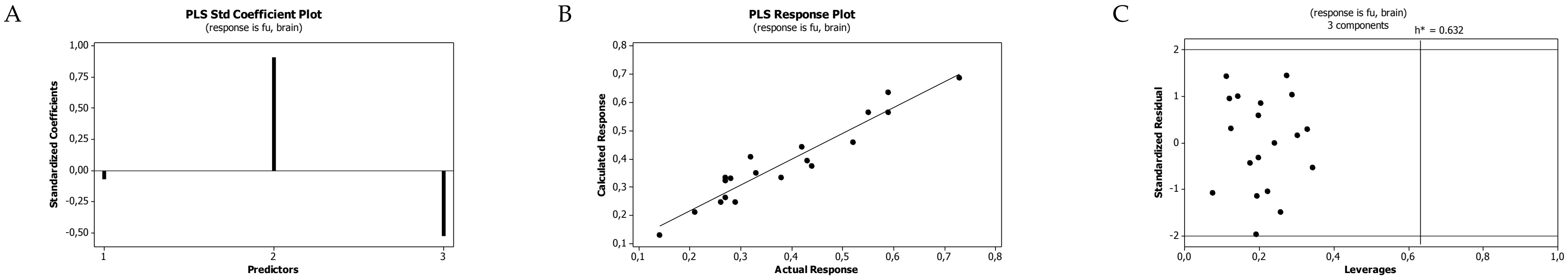

Equation (16)

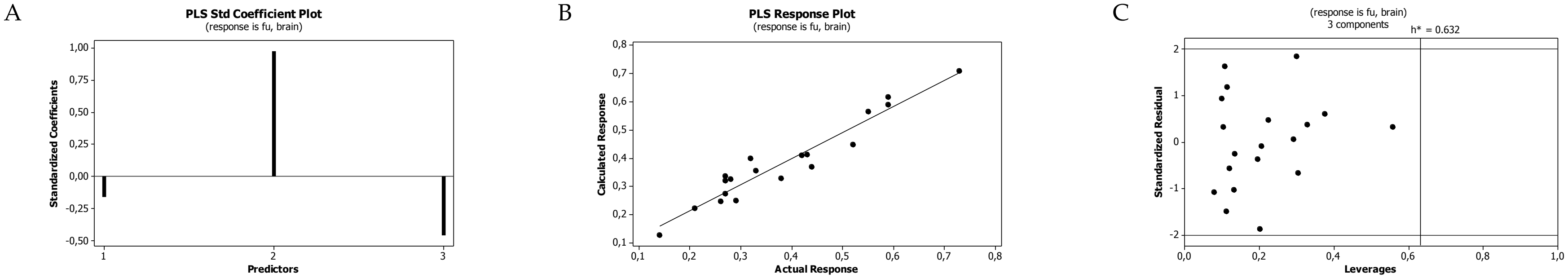

Equation (17)

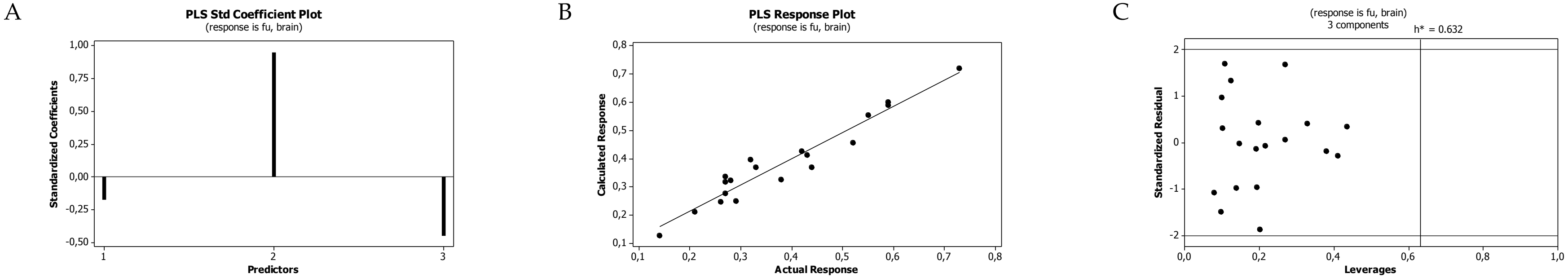

Figure S4. Standardized coefficients (A), the correlation between actual (ACD/Percepta) and predicted (Eqs 15-17) $f_{\text{u, brain}}$ parameters (B), and the Williams plots of Eqs 15-17 (C).

International Journal of *Molecular Sciences*

MDPI

Table S1. Risk assessment of adverse side effects by OSIRIS Property Explorer for the investigated compounds (1-19).

Compound	Mutagenicity	Tumorigenicity	Irritating effects	Reproductive effects
1	🟩	🟩	🟩	🟩
2	🟩	🟩	🟩	🟩
3	🟩	🟩	🟩	🟩
4	🟩	🟩	🟩	🟩
5	🟩	🟩	🟩	🟩
6	🟩	🟩	🟩	🟩
7	🟩	🟩	🟩	🟩
8	🟩	🟩	🟩	🟩
9	🟩	🟩	🟩	🟩
10	🟩	🟩	🟩	🟩
11	🟩	🟩	🟩	🟩
12	🟩	🟩	🟩	🟩
13	🟩	🟩	🟩	🟩
14	🟩	🟩	🟩	🟩
15	🟩	🟩	🟩	🟨
16	🟩	🟩	🟩	🟨
17	🟩	🟩	🟩	🟨
18	🟩	🟩	🟩	🟨
19	🟩	🟩	🟩	🟨

🟩 – no risk, score: 1.0; 🟨 – medium risk, score: 0.8
